# Supplementary material for: Genomic variation of European beech reveals signals of local adaptation despite high levels of phenotypic plasticity
Source: Nat Commun. 2024 Oct 3;15:8553. doi: 10.1038/s41467-024-52933-y (PMC11450180; doi:10.1038/s41467-024-52933-y)
Supplement: Supplementary file 3 — Reporting Summary [file 41467_2024_52933_MOESM3_ESM.pdf]

nature portfolio

Corresponding author(s): Niels A. Müller

Last updated by author(s): Aug 29, 2024

Reporting Summary

Nature Portfolio wishes to improve the reproducibility of the work that we publish. This form provides structure for consistency and transparency in reporting. For further information on Nature Portfolio policies, see our Editorial Policies and the Editorial Policy Checklist.

Statistics

For all statistical analyses, confirm that the following items are present in the figure legend, table legend, main text, or Methods section.

n/a	Confirmed	
☐	☒	The exact sample size (*n*) for each experimental group/condition, given as a discrete number and unit of measurement
☐	☒	A statement on whether measurements were taken from distinct samples or whether the same sample was measured repeatedly
☐	☒	The statistical test(s) used AND whether they are one- or two-sided<br>*Only common tests should be described solely by name; describe more complex techniques in the Methods section.*
☐	☒	A description of all covariates tested
☐	☒	A description of any assumptions or corrections, such as tests of normality and adjustment for multiple comparisons
☐	☒	A full description of the statistical parameters including central tendency (e.g. means) or other basic estimates (e.g. regression coefficient) AND variation (e.g. standard deviation) or associated estimates of uncertainty (e.g. confidence intervals)
☐	☒	For null hypothesis testing, the test statistic (e.g. *F*, *t*, *r*) with confidence intervals, effect sizes, degrees of freedom and *P* value noted<br>*Give P values as exact values whenever suitable.*
☐	☒	For Bayesian analysis, information on the choice of priors and Markov chain Monte Carlo settings
☒	☐	For hierarchical and complex designs, identification of the appropriate level for tests and full reporting of outcomes
☐	☒	Estimates of effect sizes (e.g. Cohen's *d*, Pearson's *r*), indicating how they were calculated

Our web collection on statistics for biologists contains articles on many of the points above.

Software and code

Policy information about availability of computer code

Data collection	No software was used for data collection
Data analysis	For DNA-Seq data analysis:<br>- bwa-mem version 0.7.12<br>- Picard tool's v2.26.2<br>- GATK version 4.0.5.1<br>- bcftools v1.7<br>- vcftools v0.1.16<br>- PLINK v1.9<br>- KING v2.3.0<br>- BayPass v.2.4<br>- LDBlockShow v.1.40<br><br>For data analysis and visualization:<br>- QGIS v3.32.0-Lima<br>- R v4.2.2<br>- vegan v.2.4-6<br>- LEA v3.10.1<br>- ggplot2 v3.4.2

- data.table v1.14.8
- terra v1.7-3
- geodata v0.5-8
- dplyr v.1.1.3
- patchwork v.1.1.3
- cowplot v.1.1.1
- Kendall v.2.2.1
- gradientForest v.01-37
- rrBLUP v4.6.3
- GAPIT v3.10
- vcfR v1.14.0
- rnaturalearth v1.0.1

For manuscripts utilizing custom algorithms or software that are central to the research but not yet described in published literature, software must be made available to editors and reviewers. We strongly encourage code deposition in a community repository (e.g. GitHub). See the Nature Portfolio guidelines for submitting code & software for further information.

Data

Policy information about availability of data

All manuscripts must include a data availability statement. This statement should provide the following information, where applicable:

- Accession codes, unique identifiers, or web links for publicly available datasets
- A description of any restrictions on data availability
- For clinical datasets or third party data, please ensure that the statement adheres to our policy

All DNA-seq data have been deposited in NCBI's SRA under the accession number PRJNA1005581.

Research involving human participants, their data, or biological material

Policy information about studies with human participants or human data. See also policy information about sex, gender (identity/presentation), and sexual orientation and race, ethnicity and racism.

Reporting on sex and gender	*Use the terms sex (biological attribute) and gender (shaped by social and cultural circumstances) carefully in order to avoid confusing both terms. Indicate if findings apply to only one sex or gender; describe whether sex and gender were considered in study design; whether sex and/or gender was determined based on self-reporting or assigned and methods used. Provide in the source data disaggregated sex and gender data, where this information has been collected, and if consent has been obtained for sharing of individual-level data; provide overall numbers in this Reporting Summary. Please state if this information has not been collected. Report sex- and gender-based analyses where performed, justify reasons for lack of sex- and gender-based analysis.*
Reporting on race, ethnicity, or other socially relevant groupings	*Please specify the socially constructed or socially relevant categorization variable(s) used in your manuscript and explain why they were used. Please note that such variables should not be used as proxies for other socially constructed/relevant variables (for example, race or ethnicity should not be used as a proxy for socioeconomic status). Provide clear definitions of the relevant terms used, how they were provided (by the participants/respondents, the researchers, or third parties), and the method(s) used to classify people into the different categories (e.g. self-report, census or administrative data, social media data, etc.) Please provide details about how you controlled for confounding variables in your analyses.*
Population characteristics	*Describe the covariate-relevant population characteristics of the human research participants (e.g. age, genotypic information, past and current diagnosis and treatment categories). If you filled out the behavioural & social sciences study design questions and have nothing to add here, write "See above."*
Recruitment	*Describe how participants were recruited. Outline any potential self-selection bias or other biases that may be present and how these are likely to impact results.*
Ethics oversight	*Identify the organization(s) that approved the study protocol.*

Note that full information on the approval of the study protocol must also be provided in the manuscript.

Field-specific reporting

Please select the one below that is the best fit for your research. If you are not sure, read the appropriate sections before making your selection.

[x] Life sciences   [ ] Behavioural & social sciences   [ ] Ecological, evolutionary & environmental sciences

For a reference copy of the document with all sections, see nature.com/documents/nr-reporting-summary-flat.pdf

Life sciences study design

All studies must disclose on these points even when the disclosure is negative.

Sample size	Sample size was mainly based on empirical data from other recent studies. In different poplar species (e.g. P. tremula or P. koreana), whole-genome resequencing data of 100-200 individuals from 12-24 populations was used to gain insights into the genetic basis of environmental adaptation. Theoretical power calculations strongly depend on the assumed effect sizes and population allele frequencies, but also indicate that sample sizes of several hundreds of individuals can be the required minimum. Additionally, a sufficiently high number of individuals per population is needed for reliable estimates of population allele frequencies. Since our common garden consisted of 100 populations each planted in three blocks, we sampled three trees per population and block to acquire data for 9 trees per population totaling 900 trees.
Data exclusions	Two populations (32 and 158) and one individual (B9) were excluded from the analyses, because they represent genetic outliers. Nine individuals, all from the Easternmost population from Bulgaria (provenance 158), were strongly divergent from all other individuals. The origin of this population is close to the distribution range of the second beech species in Europe, Fagus orientalis, and these individuals thus probably do not represent pure Fagus sylvatica. Additionally, the individuals from one population from Northern Germany (provenance 32) appeared admixed. Finally, one individual from a German population (individual B9) was closely related to individuals from a population from Slovakia (provenance 135) and probably represents a planting error.
Replication	Each of the 100 populations were replicated by analyzing 9 samples per population. With a single exception (individual B9, see 'data exclusion' above) all replicated samples clustered together based on genome-wide variation data. For the phenotypic data, that is stem circumference, variation within populations is much higher than variation between populations. Since beech trees cannot easily be cloned, phenotypic data are unique for each individual.
Randomization	The 100 beech populations are randomly distributed within each of the three blocks of the common garden. The nine trees sampled for each population were randomly chosen, with the only selection criterion being that their crown reaches the canopy.
Blinding	The numbering of the trees in our common gardens does not provide any biological information, therefore investigators were blinded during sampling and phenotypic data collection.

Reporting for specific materials, systems and methods

We require information from authors about some types of materials, experimental systems and methods used in many studies. Here, indicate whether each material, system or method listed is relevant to your study. If you are not sure if a list item applies to your research, read the appropriate section before selecting a response.

Materials & experimental systems

n/a	Involved in the study	
☒	☐	Antibodies
☒	☐	Eukaryotic cell lines
☒	☐	Palaeontology and archaeology
☒	☐	Animals and other organisms
☒	☐	Clinical data
☒	☐	Dual use research of concern
☐	☒	Plants

Methods

n/a	Involved in the study	
☒	☐	ChIP-seq
☒	☐	Flow cytometry
☒	☐	MRI-based neuroimaging

Dual use research of concern

Policy information about dual use research of concern

Hazards

Could the accidental, deliberate or reckless misuse of agents or technologies generated in the work, or the application of information presented in the manuscript, pose a threat to:

No	Yes	
☒	☐	Public health
☒	☐	National security
☒	☐	Crops and/or livestock
☒	☐	Ecosystems
☒	☐	Any other significant area

Experiments of concern

Does the work involve any of these experiments of concern:

No	Yes	
☒	☐	Demonstrate how to render a vaccine ineffective
☒	☐	Confer resistance to therapeutically useful antibiotics or antiviral agents
☒	☐	Enhance the virulence of a pathogen or render a nonpathogen virulent
☒	☐	Increase transmissibility of a pathogen
☒	☐	Alter the host range of a pathogen
☒	☐	Enable evasion of diagnostic/detection modalities
☒	☐	Enable the weaponization of a biological agent or toxin
☒	☐	Any other potentially harmful combination of experiments and agents

Plants

Seed stocks	Leaves were collected in May to June 2021 in the common garden in northern Germany (trial site ID: BU1901) in 96-well collection tubes.
Novel plant genotypes	No novel plant genotypes were generated in this study.
Authentication	No new seed stocks nor novel genotypes were part of this study.
