# Supplementary material for: Genomic variation of European beech reveals signals of local adaptation despite high levels of phenotypic plasticity
Source: Nat Commun. 2024 Oct 3;15:8553. doi: 10.1038/s41467-024-52933-y (PMC11450180; doi:10.1038/s41467-024-52933-y)
Supplement: Supplementary file 4 — Description of Additional Supplementary Files [file 41467_2024_52933_MOESM4_ESM.pdf]

Description of Additional Supplementary Files

File Name: Supplementary Data 1

Description: Latitude and longitude for the 100 beech populations planted in the common garden in Schädtbek (trial code: BU1901). Population numbers indicate provenance codes.

File Name: Supplementary Data 2

Description: Sequencing statistics for the 874 samples for which Illumina data were generated.

File Name: Supplementary Data 3

Description: Information on the 653 unrelated trees used for the population structure and genotype-environment association analyses. Block, row and tree indicate the position within the common garden, country the origin of the population.

File Name: Supplementary Data 4

Description: Phenotypic measurements for bud burst in 2022, bud burst in 2023 and stem circumference in winter 2022/2023 are given for the 653 unrelated trees in the common garden in Schädtbek, northern Germany (BU1901).

File Name: Supplementary Data 5

Description: Primers used for qRT-PCR of the Callose synthase 1 gene (Bhaga_2.g94).
